# Comparative Analysis of Volatile Compounds in *Tieguanyin* with Different Types Based on HS–SPME–GC–MS

**DOI:** 10.3390/foods11111530

**Published:** 2022-05-24

**Authors:** Lin Zeng, Yanqing Fu, Jinshui Huang, Jianren Wang, Shan Jin, Junfeng Yin, Yongquan Xu

**Affiliations:** 1 College of Horticulture, Fujian Agriculture and Forestry University, Fuzhou 350002, China; zenglin@tricaas.com; 2 Key Laboratory of Biology, Genetics and Breeding of Special Economic Animals and Plants, National Engineering Research Center for Tea Processing, Tea Research Institute Chinese Academy of Agricultural Sciences, Ministry of Agriculture and Rural Affairs, 9 South Meiling Road, Hangzhou 310008, China; fuyanqing@tricaas.com (Y.F.); yinjf@tricaas.com (J.Y.); 3 Anxi Taoyuan Organic Tea Farm Co., Ltd., Quanzhou 362400, China; pinya111@163.com (J.H.); pinya123@163.com (J.W.)

**Keywords:** *Tieguanyin*, oolong tea, volatile compounds, aroma

## Abstract

*Tieguanyin* (TGY) is one kind of oolong tea that is widely appreciated for its aroma and taste. To study the difference of volatile compounds among different types of TGY and other oolong teas, solid-phase microextraction–gas chromatography–mass spectrometry and chemometrics analysis were conducted in this experiment. Based on variable importance in projection > 1 and aroma character impact > 1, the contents of heptanal (1.60–2.79 μg/L), (*E*,*E*)-2,4-heptadienal (34.15–70.68 μg/L), (*E*)-2-octenal (1.57–2.94 μg/L), indole (48.44–122.21 μg/L), and (*E*)-nerolidol (32.64–96.63 μg/L) in TGY were higher than in other varieties. With the increase in tea fermentation, the total content of volatile compounds decreased slightly, mainly losing floral compounds. Heavily fermented tea contained a higher content of monoterpenoids, whereas low-fermentation tea contained higher contents of sesquiterpenes and indole, which could well distinguish the degree of TGY fermentation. Besides, the volatiles analysis of different grades of TGY showed that the special-grade tea contained more aroma compounds, mainly alcohols (28%). (*E*,*E*)-2,4-Heptadienal, (*E*)-2-octenal, benzeneacetaldehyde, and (*E*)-nerolidol were the key volatile compounds to distinguish different grades of TGY. The results obtained in this study could help enrich the theoretical basis of aroma substances in TGY.

## 1. Introduction

Oolong tea is a unique type of tea in China. Its unique floral and fruity aroma is deeply loved by consumers. Furthermore, oolong tea can improve human health because it contains rich biological functional substances, such as polyphenols, flavonoids, and amino acids. Several studies have indicated that oolong tea has the functions of anticancer, antiallergic, and improving vascular disease [1,2]. Tea variety, origin, and processing methods lead to the differences in volatile compounds among different types of oolong tea [3]. As a special tea in China, there are four famous oolong teas: Wuyi rock tea, Anxi *Tieguanyin* tea, Fenghuang Dancong tea, and Dongding oolong tea [4]. Wuyi rock tea is well known for its rich flavor and long-lasting fragrance, which is called “rock charm and floral fragrance” [5]. Fenghuang Dancong tea is well known for its unique floral and fruity aroma, which is traditionally divided into Youhua Xiang, Qilan Xiang, Yelai Xiang, etc. [6]. Anxi *Tieguanyin* tea and Dongding oolong tea have a light and elegant floral aroma. The unique biochemical composition of each cultivar greatly affects the aroma profile of oolong tea [7]. Compared with *Tieguanyin* (TGY), nitrogen exists in higher concentrations in Dongding oolong tea [8]. When choosing oolong tea varieties, higher terpenoid and green leaf volatile ratios may be a useful index for selecting cultivars [9]. The processing of oolong tea includes plucking, sun-withering, indoor-withering, shaking, fixing, rolling, and drying [10]. Aroma formation can be divided into enzymatic (before the fixing procedure) and nonenzymatic stages (after the drying procedure) [11]. During the enzymatic stage, oolong tea is formed by the hydrolysis of glycosides and carotenoids, mainly including *β*-ionone, linalool, and nerolidol [11]. During the nonenzymatic stage, the aroma compounds mainly undergo thermochemical transformation to form large amounts of heterocyclic compounds, such as furan and pyrrole [12].

At present, gas chromatography–mass spectrometry (GC–MS) combined with solid–phase microextraction (SPME) is commonly used for the analysis of tea aroma volatiles. GC–MS has a high separation effect on volatile compounds, strong identification ability, and can provide detailed information on compounds [13]. Simultaneous distillation extraction and SPME are commonly used to extract volatile compounds from tea [14]. However, volatile compounds may be degraded during the thermal processing of simultaneous distillation extraction, whereas SPME has the advantage of being fast, simple, and convenient and has been applied to wine [15], “Marion” and “Black Diamond” blackberries [16], and tea [14]. An enormous amount of data is obtained using GC–MS analysis. Principal component analysis, partial least-squares discriminant analysis (PLS–DA), and orthogonal PLS–DA can extract relevant information and discover patterns in large series of data [17], which are widely used in tea. PLS–DA is a steady discriminant statistical method that is especially suitable for cases with large numbers of explanatory variables [18,19]. Variable importance in projection (VIP) of PLS–DA can quantify the contribution of each variable to classification. The larger the VIP value, the more significant the difference in variables between different areas of oolong tea.

There are many kinds of oolong tea, among which TGY is an important one. Different varieties and fermentation degrees will lead to different flavors and qualities of TGY. In this experiment, different varieties of oolong tea were collected to analyze the difference between TGY and other varieties. The aroma difference of TGY with different grades and fermentation degrees was also analyzed. Based on SPME extraction and GC–MS analysis, nontargeted analysis was conducted on volatile aroma substances in oolong tea samples. Combined with statistical analysis, differences in aroma substances in tea samples of different varieties (TGY vs. other oolong tea), fermentation, and grades of TGY were found. Based on this study, the aroma components of TGY oolong tea with different grades and fermentation levels could be improved, and the theoretical basis of aroma substances in TGY could be enriched.

## 2. Material and Methods

### 2.1. Tea Samples

In this study, a total of 25 tea samples were collected (Figure S1), including five special-grade TGY with low fermentation (LF-T), five special-grade TGY with heavy fermentation (HF-T), five first-grade TGY with heavy fermentation (HF-F), five other TGY samples, two *Huangdan* samples (HD), one *Baiyaqilan* sample (BYQL), and two *Zhangpinshuixian* samples (ZPSX). All tea samples were purchased from the local markets in Fujian, China. All tea samples were sealed in containers and stored in a −20 °C freezer for further analysis.

### 2.2. Chemicals and Instruments

Decanoic acid ethyl ester (analytically pure reagent, purity ≥ 99.5%) was purchased from Shanghai Guo Yao Group Chemical Reagent Co., Ltd. (Shanghai, China). Purified water used in this experiment was purchased from Hangzhou Wahaha Group Co., Ltd. (Hangzhou, China). A standard mixture of n-alkanes C8–C30 was purchased from o2si (North Charleston, SC, USA).

### 2.3. Tea Aroma Extraction Using SPME

The fiber was preconditioned for 5 min in the injection port of the gas chromatograph at 230 °C to remove any volatiles remaining on the fiber before each extraction. Tea samples (0.1 g) were weighed and placed in one 20 mL headspace vial, then 5 mL of boiling distilled water and 20 μL of decanoic acid ethyl ester (5 μg/L internal standard) were added. The vials were kept in a 60 °C water bath for 5 min. After that, the SPME fiber was used for the extraction of volatiles for 60 min in a 60 °C water bath. Subsequently, the volatiles were desorbed at the injector (230 °C) of the GC–MS for 5 min [20].

### 2.4. GC–MS Analysis of Volatile Compounds

An Agilent 6890 gas chromatograph interfaced with an Agilent HP 5973 MSD ion trap mass spectrometer (Wilmington, DE, USA) was used for the analysis of volatiles. The separation was performed on a DB-5MS capillary column (30 m × 250 μm × 0.25 μm). The GC inlet temperature was set at 230 °C. High purity helium (99.999%) was used as the carrier gas with a constant flow of 0.544 mL/min. The temperature procedure was as follows: 40 °C for 3 min, raised to 120 °C at 2 °C/min, then held at 120 °C for 2 min, and finally raised to 230 °C at 10 °C/min and held for 2 min. For MS analysis, the electronic energy of the EI mode was 70 eV. The temperature of the ion source was set at 230 °C. The mass scan range was *m/z* 40–400. Each sample was analyzed in triplicate [21].

### 2.5. Statistical Analysis

The volatile compounds were identified using retention indices (RIs), authentic standards, or comparison with mass spectra in the National Institute of Standards and Technology library (NIST14.L). The linear RIs were determined via sample injection with a homologous series of alkanes (C_5_−C_30_) (Sigma-Aldrich (Shanghai, China)). The PLS-DA was performed using SIMCA-P 13.0 software (Umetric, Umea, Sweden). MultiExperiment Viewer software (version 4.7.4, Boston, MA, USA) was employed for heatmap analysis. ACI value calculation reference [22,23] as a standard.

## 3. Results and Discussion

### 3.1. Identification of Volatile Compounds in TGY

**Figure 1:**
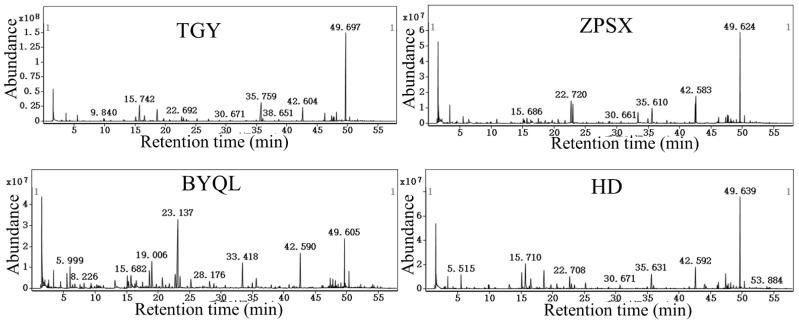
GC–MS total ion chromatogram of aroma components in the four tea varieties sampled.

Volatile compounds obtained using GC–MS analysis (Figure 1) were identified using NIST14.L, combined with the retention time, indices, reference data, and data processing software. Finally, a total of 118 volatile compounds were identified, namely 18 alcohols, 13 aromatics, 23 aldehydes, 10 ketones, 18 heterocyclic compounds, 5 N-containing compounds, 22 esters, and 9 other compounds. The relative content of the identified compounds was calculated according to internal standards [20]. The analysis results showed that the main volatile compounds of TGY were (*E*)-nerolidol, indole, (*E*,*E*)-2,4-heptadienal, benzeneacetaldehyde, hotrienol, linalool, n-butyl acetate, hexanal, and phenylethyl alcohol. Among them, (*E*)-nerolidol (11.86–21.14%), indole (12.15–34.05%), and (*E*,*E*)-2,4-heptadienal (6.13–18.12%) were the three most abundant volatile compounds with the highest content in TGY samples, which was consistent with the results of previous studies [24,25]. Retention time, odor description, and type of volatile compounds are listed in Table 1.

### 3.2. Differences of Volatile Compounds in TGY from Other Varieties of Oolong Tea

**Figure 2:**
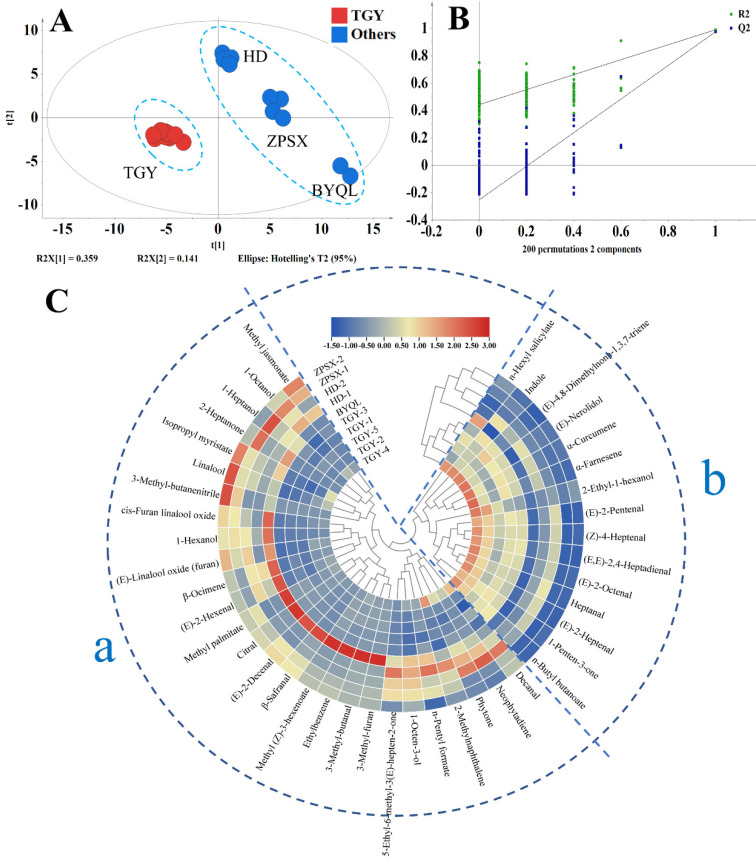
GC−MS analysis results of *Tieguanyin* and other varieties. (**A**) The score scatter plots of PLS−DA of TGY and four other varieties. (**B**) Validation of the PLS−DA model. (**C**) Heatmap of differential substances in different varieties. TGY: *Tieguanyin*, HD: *Huangdan*, BYQL: *Baiyaqilan*, ZPSX: *Zhangpinshuixian*. Figure 2B: The vector value of R2 (0.0, 0.445) and Q2 (0.0, −0.251) from 200permutations, which indicated that this PLS−DA model was not overfitting. Figure 2C: The contents of compounds in group a were lower in TGY, the content of compounds in group b was higher in TGY.

The data obtained using GC–MS analysis were analyzed with PLS–DA after data preprocessing. Figure 2A shows that there is clear discrimination between TGY and other varieties of oolong tea; HD, ZPSX, and BYQL could also be clearly separated. The PLS–DA model was confirmed by 200 permutation tests (Figure 2B). The results indicated that the model was not overfitted. Not all identified volatile compounds played an important role in the differentiation analysis of different types of oolong tea samples. To find the key differential volatiles, after PLS-DA analysis, compounds with VIP > 1 were screened out for further analysis (Figure 2C). Compounds with VIP > 1 were generally considered to be the important contributors to tea aroma difference. These compounds were divided into two groups (a and b in Figure 2C). The contents of compounds in group a were lower in TGY, including methyl jasmonate, 1-octanol, linalool, and its oxides. Methyl jasmonate has a powerful floral-herbaceous and sweet aroma, linalool has a floral aroma, and 1-octanol presents a penetrating aromatic aroma. This may be the reason why other varieties were sweeter than TGY. In group b, the content of compounds in TGY was higher, mainly including (*E*)-nerolidol, indole, and α-farnesene. These aromatic compounds were characteristic of oolong tea aroma [11]. Cluster analysis could distinguish TGY samples from other tea variety samples, and HD, ZPSX, and BYQL were also separated. This indicates that variety selection was very important to the aroma characteristics of oolong tea.

There were still many differential compounds screened by PLS-DA. Aroma character impact (ACI) was introduced to further screen the differential compounds. ACI is a ratio of odor-activity in a mixture and is more useful for comparing the contribution of the individual components to the overall aroma [22,23]. Therefore, ACI values of compounds (VIP > 1) were calculated, and the results are shown in Table 2. The contents of heptanal, (*E*,*E*)-2,4-heptadienal, (*E*)-2-octenal, indole, and (*E*)-nerolidol in TGY were higher than in other varieties, whereas the content of 1-octen-3-ol and linalool were lower. (*E*,*E*)-2,4-Heptadienal as fatty and oil notes, was mostly derived from lipid degradation during manufacture [26] and contained a larger quantity in high-grade green or black tea [27]. (*E*)-2-Octanal has a fatty, green aroma. Indole is widely distributed and plays an important role in plants and accumulates at the turnover stage of the oolong tea manufacturing process [28]. (*E*)-Nerolidol is a sesquiterpene presenting as an essential oil in many plants with a floral odor [29] and as a potent signal that elicits plant defenses [30]. The proportion of indole and (*E*)-nerolidol were higher in TGY, which might be caused by its fragrant and fruit aroma. 1-Octen-3-ol has a sweet earthy odor and is often used as mosquito bait [31]. Linalool is a mate attractant pheromone component in the bee *Colletes cunicularius* with a floral aroma [32]. Taken together, the data indicate that (*E*,*E*)-2,4-heptadienal, (*E*)-2-octenal, indole, (*E*)-nerolidol, 1-octen-3-ol, and linalool were key differentiating volatiles of TGY from other varieties.

### 3.3. Difference Analysis of Volatiles in TGY with Different Fermentation

Oolong tea fermentation mainly occurs in the withering and turnover procedures. In the fermentation process, grassy flavors were diminished, and the flowery and fruity fragrances appeared sequentially [24]. The reason was that the continuous mechanical damage during fermentation facilitated the synthesis of terpenoids, fatty acids, and benzenoid-derived compounds [34], such as trans-*β*-ocimene, indole, and linalool [35]. Therefore, the degree of fermentation was very important to the quality of oolong tea.

**Figure 3:**
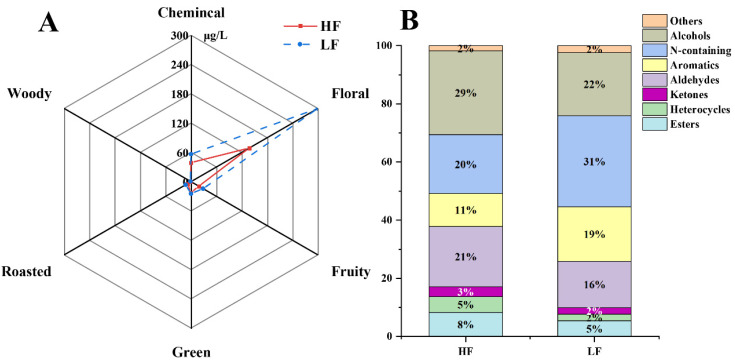
(**A**) Composition proportion of aroma of *Tieguanyin* with different fermentations. (**B**) Proportion of aroma types of *Tieguanyin* with different fermentations. HF: heavy fermentation, LF: low fermentation.

In this study, 118 volatile compounds were identified by analyzing TGY samples of different fermentation levels and classified according to aroma type and compound type (Figure 3A,B), the floral and fruity compounds were dominant in TGY. With the continuation of fermentation, the total content of compounds decreased, mainly the floral aroma compounds. The compounds with the highest proportion in HF-T were alcohols, whereas that in LF-T were N-containing compounds. Aldehydes and alcohols were often characterized by experts with strong sensory descriptions and associated with greenery, freshness, green plants, citrusy, fatty, and sweet notes [26].

**Figure 4:**
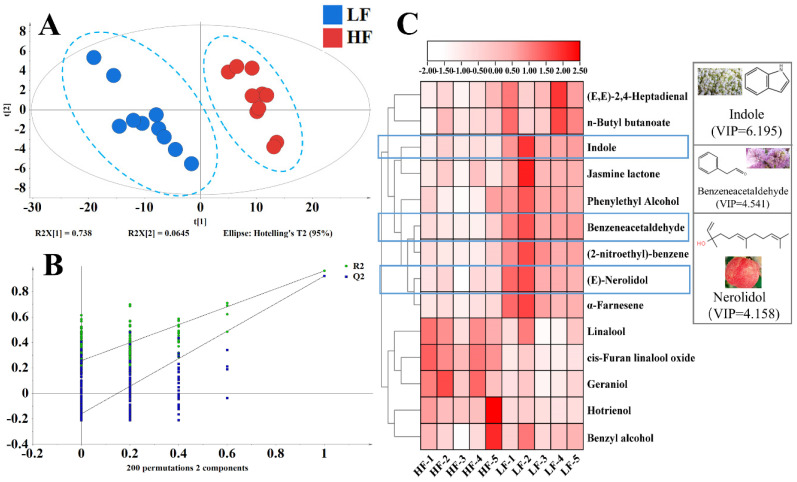
GC−MS analysis results of differently fermented *Tieguanyin*. (**A**) The score scatter plots of PLS−DA of TGY. (**B**) Validation of the PLS−DA model. (**C**) Heatmap of differential substances in different fermentation *Tieguanyin.* HF: heavy fermentation, LF: low fermentation. Figure 4B: The vector value of R2 (0.0, 0.258) and Q2 (0.0, −0.16) from 200permutations, which indicated that this PLS−DA model was not overfitting.

Through data analysis, the TGY samples with different fermentation levels were clearly separated in the PLS-DA plot (Figure 4A). To eliminate the interference of irrelevant variables and find the key compounds that affected this classification of tea samples, VIPs were used to screen compounds with significant differences among different fermentations of TGY. As the fermentation level increased (Figure 4C), the contents of (*E*,*E*)-2,4-heptadienal, *n*-butyl butanoate, indole, jasmine lactone, phenylethyl alcohol, benzeneacetaldehyde, (2-nitroethyl)-benzene, (*E*)-nerolidol, and *α*-farnesene decreased, whereas the content of hotrienol, benzyl alcohol, geraniol, linalool, and its oxides increased, which was consistent with previous studies [36]. Hotrienol, geraniol, and linalool are monoterpenoids, which were induced and composed by the methylerythritol phosphate pathway. (*E*)-Nerolidol and α-farnesene are sesquiterpenes and were induced and composed by the mevalonic acid pathway [37]. The synthesis of these terpenes requires the same precursor, geranyl pyrophosphate, and there may be competition between the two pathways. The mevalonic acid pathway may be dominant when the fermentation degree is low. Monoterpenes were synthesized mainly through the methylerythritol phosphate pathway at high fermentation levels. The content of indole was high in lightly fermented oolong tea, but low in heavily fermented Beauty tea or black tea [10,28], which was consistent with our study results that indole content decreased with the deepening of fermentation. In conclusion, HF-T contained a high content of monoterpenoids, whereas LF-T contained a high content of sesquiterpenes and indole. These compounds were useful for the classification of TGY with different fermentation degrees.

### 3.4. Difference Analysis of Volatiles in Different Grades of TGY

**Figure 5:**
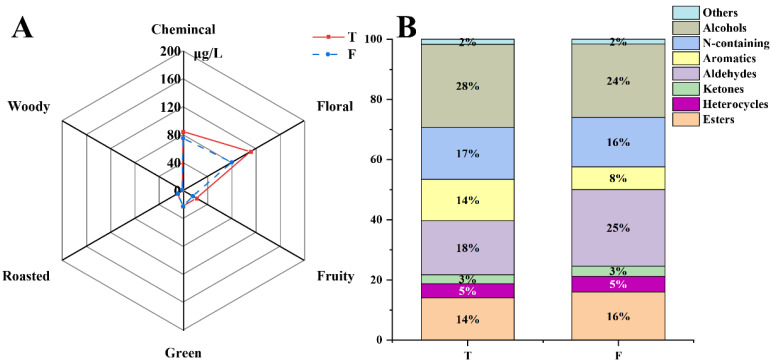
(**A**) Composition proportion of TGY aromas in different grades. (**B**) Proportion of aroma types of TGY in different grades. T: special grade, F: first grade.

According to the tenderness, aroma, taste, and appearance, different types of tea can be classified into different grades [38]. TGY is usually classified into special grades and grades 1–4 [39]. Exploring the signature compounds of different grades of TGY could help identify the grade of TGY. Then, in this study, the differences in volatiles of TGY with different grades were analyzed. As shown in Figure 5, the total amount of aroma in the special-grade tea was higher than that in first-grade tea, especially in the floral-scented compounds. Therefore, the special-grade tea was richer in floral types under sensory evaluation, which was consistent with previous studies [27,40]. Based on the analysis of compound types, the highest proportion of volatile compounds in the special grade tea was alcohols (28%). In first-grade tea, aldehydes accounted for a higher proportion (25%), which may be caused by the oxidation of more primary alcohols into aldehydes.

**Figure 6:**
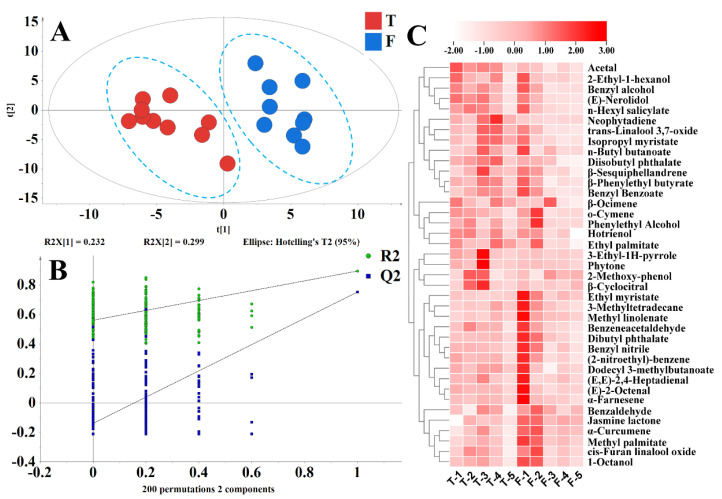
GC–MS analysis results of different grades of *Tieguanyin*. (**A**) The score scatterplots of PLS–DA of TG. (**B**) Validation of the PLS–DA model. (**C**) Heatmap of differential substances in different grades. T: special grade, F: first grade. Figure 6B: The vector value of R2 (0.0, 0.561) and Q2 (0.0, −0.138) from 200permutations, which indicated that this PLS−DA model was not overfitting.

The PLS–DA analysis result is shown in Figure 6. Volatile compounds with VIP > 1 were screened out (Figure 6C). Compared with the special grade tea, the relative content of benzaldehyde (volatile oil of almond), jasmine lactone (coconut-fruity odor), and *α*-farnesene (woody, citrus, sweet) in first-grade tea were higher, but that of acetal (pleasant odor), 2-ethyl-1-hexanol (mild, oily, sweet, slightly floral odor), benzyl alcohol (faint, aromatic, fruity odor), (*E*)-nerolidol (rose apple), and *n*-hexyl salicylate is lower. (*E*)-Nerolidol content was positively correlated with oolong tea grade [41,42]. The special-grade tea and first-grade tea were the highest grades of tea, and their quality evaluation was used to find out whether there was an inferior odor in the tea aroma and whether the aroma type was typical. For example, the fresh-scented TGY typically had a fresh flowers aroma, whereas that of Oriental Beauty was honey and sweet aroma. The aroma of benzaldehyde had a roasted aroma, which was not consistent with the TGY aroma type.

Here, ACI values were also calculated to further screen out key aroma compounds related to TGY grades (Table 3). The content of (*E*,*E*)-2,4-heptadienal (1.68–2.19%) and (*E*)-2-octenal (6.36–9.10%) was higher in first-grade tea, whereas the content of benzeneacetaldehyde (0.83–1.25%) and (*E*)-nerolidol (0.06–0.1%) was lower. (*E*,*E*)-2,4-Heptadienal had a fatty and oil aroma, and its concentration was lower in the special grade tea, which was opposite to the previous results [27]. (*E*)-2-Octenal had a fatty and green aroma, and gave rise to inferior flavor. (*E*)-Nerolidol was an important contributor to oolong tea aroma, which could be regarded as one of the key odors of oolong tea quality. In general, its content was positively correlated with oolong tea grade [42]. In conclusion, the higher grade was the grade of TGY, the more volatile compounds that were present. Furthermore, (*E*,*E*)-2,4-heptadienal, (*E*)-2-octenal, benzeneacetaldehyde, and (*E*)-nerolidol could be used as the key volatile compounds to distinguish different grades of TGY.

## 4. Conclusions

In this study, a combination of SPME–GC–MS and chemometrics analysis provided a convenient and reproducible method for differential analysis of oolong tea samples. The content of heptanal, (*E*,*E*)-2,4-heptadienal, (*E*)-2-octenal, indole, and (*E*)-nerolidol in TGY was higher than in other varieties, whereas the content of 1-octen-3-ol and linalool was lower than in other varieties. With the extension of fermentation, HF contains a high content of monoterpenoids, whereas LF contains a high content of sesquiterpenes and indole. (*E*,*E*)-2,4-Heptadienal, (*E*)-2-octenal, benzeneacetaldehyde, and (*E*)-nerolidol were the key volatile compounds to distinguish different grades of TGY. (*E*)-nerolidol, (*E*,*E*)-2,4-heptadienal, and (*E*)-2-octanal were important compounds contributing to the aroma quality of TGY. The results enriched the theoretical basis of aroma substances in TGY and could also provide theoretical guidance for consumers to choose tea.

## Figures and Tables

**Table 1:** Identified volatile compounds in *Tieguanyin*.

Retention Time	Volatile Compounds	RI	ID ^a^	Odor Type	Odor Description ^b^
2.071	3-Methyl-furan	594	MS, RI	Roasted	/
2.385	Acetic acid	613	RI	Chemical	Strong odor of vinegar
2.516	3-Methyl-butanal	621	MS, RI	Fruity	Apple-like
2.624	2-Methyl-butanal	627	MS, RI	Roasted	/
2.856	1-Penten-3-ol	641	MS, RI	Green	Grassy-green
2.912	1-Penten-3-one	645	MS, RI	Chemical	Penetrating
3.060	Pentanal	654	MS, RI	Chemical	Strong, acrid, pungent odor
3.124	2-Ethyl-furan	657	MS, RI	Roasted	Smoky burnt
3.709	3-Methyl-butanenitrile	693	MS, RI	/	/
3.714	Acetal	693	MS, RI	Floral	Pleasant odor
4.008	2-Methyl-butanenitrile	711	RI	/	odorless
4.244	(*E*)-2-Pentenal	725	MS, RI	Green	Pungent green
4.514	Toluene	741	MS, RI	Chemical	Benzene-like
4.709	(*Z*)-2-Penten-1-ol	753	MS, RI	Green	Green diffusive
5.520	Hexanal	801	MS, RI	Green	Strong, green
5.521	n-Butyl acetate	801	RI	Fruity	Fruity
6.015	3-Ethyl-1H-pyrrole	812	MS, RI	Roasted	/
6.414	2-Ethyl-2-butenal	821	RI	Green	Grassy green
6.556	n-Pentyl formate	824	RI	Fruity	Plum-like
6.815	Furfural	830	MS, RI	Roasted	Almond-like
7.600	(*E*)-2-Hexenal	848	MS, RI	Green	Vegetable-like
7.868	Ethylbenzene	855	MS, RI	Floral	Aromatic
8.237	1,3-Dimethyl-benzene	863	MS, RI	Floral	Sweet
8.464	1-Hexanol	868	MS, RI	Green	Sweet alcohol
9.264	Styrene	887	RI	Floral	Floral
9.456	2-Heptanone	891	MS, RI	Fruity	Fruity
9.849	(*Z*)-4-Heptenal	900	MS, RI	Green	Fatty, green
9.951	Heptanal	902	MS, RI	Green	Penetrating fruity
10.547	Acetylfuran	912	MS, RI	Roasted	Coffee-like
11.353	Methyl hexoate	925	RI	Fruity	Pineapple
11.353	Methyl (*Z*)-3-hexenoate	925	MS, RI	Fruity	Fruity
13.092	(*E*)-2-Heptenal	954	MS, RI	Green	Pungent green
13.147	Benzaldehyde	955	MS, RI	Roasted	Almond
13.671	5-Methyl-2-furancarboxaldehyde	963	MS, RI	Roasted	Caramellic
14.148	1-Heptanol	971	MS, RI	Green	Fragrant
14.666	1-Octen-3-ol	980	MS, RI	Chemical	Sweet earthy
15.114	6-Methyl-5-Hepten-2-one	987	MS, RI	Green	Green citrus-like
15.297	*β*-Myrcene	990	MS, RI	Woody	/
15.678	(*E*,*E*)-2,4-Heptadienal	996	MS, RI	Chemical	Fatty, green
15.774	*n*-Butyl butanoate	998	RI	Fruity	Fruity, pineapple-
16.082	Octanal	1003	MS, RI	Fruity	Strong, fruity
17.025	1,2,3-Trimethyl-benzene	1016	MS, RI	Chemical	Aromatic
17.284	*o*-Cymene	1020	MS, RI	Floral	Aromatic
17.528	D-Limonene	1024	MS, RI	Fruity	Citrus odor
17.720	1,3-Dichloro-benzene	1027	RI	Floral	Aromatic
17.941	2-Ethyl-1-hexanol	1030	MS, RI	Floral	Floral
18.197	Benzyl alcohol	1034	MS, RI	Fruity	Faint aromatic
18.607	Benzeneacetaldehyde	1040	MS, RI	Floral	Green floral and sweet
19.000	1-Ethyl-2-formylpyrrole	1046	MS, RI	Roasted	burnt smokey
19.087	*β*-Ocimene	1047	MS, RI	Floral	/
19.739	(*E*)-2-Octenal	1056	MS, RI	Green	Fatty, green aroma
20.105	Acetophenone	1062	RI	Fruity	Oranges
20.652	*cis*-Furan linalool oxide	1070	MS, RI	/	/
20.847	1-Octanol	1073	MS	Floral	Penetrating Aromatic
21.755	(*E*)-Linalool oxide (furan)	1086	MS, RI	Floral	/
21.790	2-Methoxy-phenol	1087	RI	Roasted	Smoky
22.729	Linalool	1100	MS, RI	Floral	Floral odor
23.020	Hotrienol	1105	MS, RI	Floral	Mouldy
23.451	Phenylethyl Alcohol	1111	MS, RI	Fruity	Honey-like
23.805	(*E*)-4,8-Dimethylnona-1,3,7-triene	1116	RI	/	/
25.190	Benzyl nitrile	1136	MS, RI	Floral	Aromatic
25.882	5-Ethyl-6-methyl-3(*E*)-hepten-2-one	1146	RI	/	/
27.343	*trans*-Linalool 3,7-oxide	1167	MS, RI	/	/
28.265	Octanoic acid	1180	RI	Chemical	Unpleasant
28.733	*α*-Terpineol	1187	MS, RI	Floral	Pleasant, floral
28.736	1-Furfurylpyrrole	1187	MS, RI	Roasted	Vegetable aroma
28.864	Methyl salicylate	1189	MS, RI	Green	Wintergreen
29.059	*trans*-3,7-Dimethyl-1,5-octadien-3,7-diol	1192	MS, RI	/	/
29.257	*β*-Safranal	1195	MS, RI	Green	Green-floral
29.969	Decanal	1205	MS, RI	Floral	Floral-fatty odor
30.205	2,4-Dimethyl-benzaldehyde	1208	MS, RI	Roasted	Bitter-almond
30.671	*β*-Cyclocitral	1215	MS, RI	Woody	/
31.828	(*3Z*)-3-Hexenyl 2-methylbutanoate	1233	RI	/	/
32.174	Isovaleric acid, dodecyl ester	1238	RI	Fruity	Fruity
33.137	*β*-Cyclohomocitral	1252	MS, RI	/	/
33.370	Geraniol	1256	MS, RI	Floral	Sweet rose odor
33.693	(*E*)-2-Decenal	1260	MS, RI	Green	Green, fatty
34.313	Citral	1270	MS, RI	Fruity	Strong lemon
35.648	Indole	1290	MS, RI	Floral	Light jasmine
35.982	(2-nitroethyl)-benzene	1294	MS, RI	/	/
36.201	2-Methylnaphthalene	1298	RI	/	/
40.331	2-Undecenal	1362	MS, RI	Fruity	Orange peel
40.808	3-hydroxy-2,2,4-trimethylpentyl isobutyrate	1370	MS, RI	/	Characteristic
41.568	*β*-Damascenone	1382	MS, RI	Fruity	Floral, fruity
41.585	*cis*-3-Hexenyl hexanoate	1382	MS, RI	Green	Fruity green
41.917	*n*-Hexyl hexanoate	1387	MS, RI	Green	Herbaceous
42.467	Jasmone	1396	MS, RI	Floral	Odor of jasmine
42.702	Dodecanal	1399	RI	Chemical	Fatty
44.032	Syrfynol 104	1425	MS, RI	/	/
44.279	*α*-Ionone	1430	MS, RI	Floral	/
45.256	*β*-Phenylethyl butyrate	1448	MS, RI	Fruity	/
45.422	Octyl-cyclohexane	1452	RI	/	/
46.481	3-Methyltetradecane	1472	RI	/	/
47.168	1-Dodecanol	1485	RI	Fruity	Sweet
47.220	*α*-Curcumene	1486	RI	/	/
47.357	2,6-Di-tert-butylbenzoquinone	1489	RI	/	/
47.607	Jasmine lactone	1494	MS, RI	Roasted	Coconut-fruity
48.166	*α*-Farnesene	1509	MS, RI	Fruity	Citrus, herbal, lavender-like
48.355	2,4-Di-tert-butylphenol	1517	MS, RI	/	/
48.512	*β*-Sesquiphellandrene	1524	MS, RI	/	/
49.664	(*E*)-Nerolidol	1571	MS, RI	Floral	Rose apple
50.324	Txib	1599	MS, RI	Chemical	Musty
50.342	Cedrol	1599	RI	Fruity	Cedar-like
51.264	Methyl jasmonate	1654	MS, RI	Floral	Powerful floral-herbaceous, sweet aroma
51.704	*n*-Hexyl salicylate	1680	MS, RI	/	/
53.016	Benzyl Benzoate	1772	MS, RI	Floral	Faint, pleasant
53.339	Ethyl myristate	1796	MS, RI	Chemical	Waxy
53.729	Isopropyl myristate	1828	MS, RI	/	Odorless
53.883	Neophytadiene	1842	MS, RI	/	/
53.970	Phytone	1849	MS, RI	/	/
54.084	Caffeine	1859	MS, RI	/	Odorless
54.287	Diisobutyl phthalate	1876	MS, RI	Chemical	Slight ester
54.872	Methyl palmitate	1926	MS, RI	Chemical	Oily, waxy, fatty
54.898	7,9-Di-tert-butyl-1-oxaspiro (4,5) deca-6,9-diene-2,8-dione	1928	MS, RI	/	/
55.329	Dibutyl phthalate	1965	MS, RI	Floral	Slight, aromatic
55.591	Hexadecanoic acid, ethyl ester	1987	MS, RI	Floral	Slight, aromatic
56.725	Methyl linolenate	2083	MS, RI	/	/
56.929	Phytol	2101	MS, RI	Floral	Floral, balsam, powdery, waxy

‘/’, information was not found in the literature. ^a^: Identification methods. MS, identification based on the NIST14.L; RI, retention index. ^b^: Odor description found in the literature with database (Flavornet; https://pubchem.ncbi.nlm.nih.gov/ (accessed on 10 January 2022).

**Table 2:** The key compounds associated with *Tieguanyin* and other varieties with significantly high odor-activity values (VIP > 1).

Volatile Compounds	ACI (%)										OT (μg/L)
	TGY-1	TGY-2	TGY-3	TGY-4	TGY-5	HD-1	HD-2	BYQL	ZPSX-1	ZPSX-2	
3-Methyl-butanal	0.02	0.03	0.02	0.03	0.03	0.06	0.06	0.52	0.08	0.08	1.1
1-Penten-3-one	0.10	0.10	0.08	0.09	0.09	0.04	0.07	0.02	0.02	0.03	23
(*E*)-2-Pentenal	0.00	0.00	0.00	0.00	0.00	0.00	0.00	0.00	0.00	0.00	980
(*E*)-2-Hexenal	0.01	0.01	0.01	0.01	0.01	0.02	0.03	0.04	0.02	0.02	19.2
Ethylbenzene	0.00	0.00	0.00	0.00	0.00	0.00	0.00	0.00	0.00	0.00	220.5
1-Hexanol	0.01	0.01	0.01	0.01	0.01	0.02	0.03	0.06	0.04	0.03	5.6
2-Heptanone	0.00	0.00	0.00	0.00	0.00	0.00	0.00	0.00	0.01	0.00	140
(*Z*)-4-Heptenal	0.00	0.00	0.00	0.00	0.00	0.00	0.00	0.00	0.00	0.00	900
Heptanal	0.54	0.60	0.53	0.66	0.51	0.36	0.54	0.19	0.21	0.19	2.8
Methyl (*Z*)-3-hexenoate	0.00	0.00	0.00	0.00	0.00	0.00	0.00	0.00	0.00	0.00	70
(*E*)-2-Heptenal	0.19	0.21	0.17	0.22	0.16	0.13	0.17	0.09	0.08	0.07	2.8
1-Heptanol	0.00	0.01	0.01	0.01	0.01	0.02	0.02	0.02	0.05	0.01	5.4
1-Octen-3-ol	0.32	0.34	0.34	0.39	0.34	0.74	0.67	0.67	0.65	0.42	1.5
(*E*,*E*)-2,4-Heptadienal	2.46	2.84	2.60	3.15	1.93	1.16	1.99	0.61	0.26	0.24	15.4
2-Ethyl-1-hexanol	0.00	0.00	0.00	0.00	0.00	0.00	0.00	0.00	0.00	0.00	25,480
*β*-Ocimene	0.01	0.01	0.01	0.01	0.01	0.02	0.02	0.04	0.01	0.01	34
(*E*)-2-Octenal	8.51	8.86	8.27	10.14	6.45	5.18	6.92	3.13	2.99	2.86	0.2
*cis*-Furan linalool oxide	0.00	0.00	0.00	0.00	0.00	0.00	0.00	0.01	0.00	0.00	320
1-Octanol	0.00	0.00	0.00	0.00	0.00	0.00	0.00	0.00	0.00	0.00	125.8
(*E*)-Linalool oxide (furan)	0.00	0.00	0.00	0.00	0.00	0.00	0.00	0.00	0.00	0.00	320
Linalool	17.95	21.60	17.42	22.36	20.35	22.35	26.56	25.49	29.38	39.53	0.22
*β*-Safranal	0.01	0.02	0.02	0.02	0.02	0.04	0.04	0.23	0.10	0.10	3
Decanal	0.04	0.03	0.03	0.05	0.03	0.04	0.06	0.04	0.04	0.04	3
(*E*)-2-Decenal	0.15	0.10	0.19	0.18	0.11	0.16	0.11	2.84	1.31	1.35	0.4
Citral	0.00	0.00	0.00	0.00	0.00	0.00	0.00	0.03	0.01	0.01	400
Indole	1.35	1.34	1.79	1.97	1.39	1.60	0.55	0.22	0.71	0.44	40
*α*-Farnesene	0.05	0.04	0.04	0.06	0.03	0.03	0.02	0.02	0.01	0.01	87
(*E*)-Nerolidol	0.20	0.16	0.18	0.25	0.13	0.17	0.07	0.03	0.07	0.05	250
Methyl jasmonate	0.01	0.01	0.01	0.01	0.01	0.03	0.01	0.01	0.04	0.04	3
*n*-Hexyl salicylate	0.00	0.00	0.00	0.00	0.00	0.00	0.00	0.00	0.00	0.00	73
Methyl palmitate	0.00	0.00	0.00	0.00	0.00	0.00	0.00	0.00	0.00	0.00	19,000

OT: odor thresholds in water were obtained from [33]. TGY: *Tieguanyin*, HD: *Huangdan*, BYQL: *Baiyaqilan*, ZPSX: *Zhangpinshuixian*. Aroma character impact (ACI): a ratio of odor-activity in a mixture and is more useful for comparing the contribution of the individual components to the overall aroma.

**Table 3:** The key compounds responsible for the different grades of TGY with significantly high odor-activity values (VIP > 1).

Volatile Compounds	ACI (%)										OT (μg/L)
	T-1	T-2	T-3	T-4	T-5	F-1	F-2	F-3	F-4	F-5	
Acetal	0.00	0.00	0.00	0.00	0.00	0.00	0.00	0.00	0.00	0.00	80
3-Ethyl-1H-pyrrole	0.00	0.00	0.00	0.00	0.00	0.00	0.00	0.00	0.00	0.00	10,000
Benzaldehyde	0.00	0.00	0.00	0.00	0.00	0.00	0.00	0.00	0.00	0.00	750.89
(*E*,*E*)-2,4-Heptadienal	1.23	1.64	2.09	1.44	1.26	2.19	2.17	1.68	2.05	2.00	15.4
*o*-Cymene	0.06	0.06	0.06	0.04	0.03	0.06	0.04	0.03	0.03	0.03	11.4
2-Ethyl-1-hexanol	0.00	0.00	0.00	0.00	0.00	0.00	0.00	0.00	0.00	0.00	25,480
Benzyl alcohol	0.00	0.00	0.00	0.00	0.00	0.00	0.00	0.00	0.00	0.00	254.6
Benzeneacetaldehyde	2.19	1.39	3.20	3.9	2.25	1.25	0.90	0.83	1.08	1.14	6.3
*β*-Ocimene	0.01	0.01	0.01	0.01	0.01	0.01	0.01	0.01	0.00	0.00	34
(*E*)-2-Octenal	5.46	6.31	7.21	5.77	4.91	9.10	7.88	6.36	7.58	6.83	0.2
*cis*-Furan linalool oxide	0.01	0.01	0.01	0.01	0.01	0.01	0.01	0.01	0.01	0.01	320
1-Octanol	0.00	0.00	0.00	0.00	0.00	0.00	0.00	0.00	0.00	0.00	125.8
Hotrienol	0.08	0.06	0.16	0.06	0.06	0.06	0.06	0.05	0.06	0.04	110
Phenylethyl alcohol	0.01	0.00	0.01	0.01	0.01	0.00	0.00	0.00	0.00	0.00	564
*trans*-Linalool 3,7-oxide	0.00	0.00	0.00	0.00	0.00	0.00	0.00	0.00	0.00	0.00	190
*β*-Cyclocitral	0.14	0.19	0.17	0.14	0.13	0.26	0.22	0.17	0.17	0.17	3
*β*-Phenylethyl butyrate	0.00	0.00	0.00	0.00	0.00	0.00	0.00	0.00	0.00	0.00	376
*α*-Farnesene	0.01	0.02	0.02	0.03	0.02	0.02	0.01	0.02	0.02	0.02	87
(*E*)-Nerolidol	0.10	0.13	0.12	0.17	0.11	0.06	0.07	0.10	0.09	0.09	250
*n*-Hexyl salicylate	0.00	0.00	0.00	0.00	0.00	0.00	0.00	0.00	0.00	0.00	73
Benzyl Benzoate	0.00	0.00	0.00	0.00	0.00	0.00	0.00	0.00	0.00	0.00	341
Methyl palmitate	0.00	0.00	0.00	0.00	0.00	0.00	0.00	0.00	0.00	0.00	19,000

OT: odor thresholds in water were obtained from [33]. Aroma character impact (ACI): a ratio of odor-activity in a mixture and is more useful for comparing the contribution of the individual components to the overall aroma.

## Data Availability

The data presented in this study are available on request from the corresponding author.

## Supplementary Materials

The following supporting information can be downloaded at: https://www.mdpi.com/article/10.3390/foods11111530/s1. Figure S1: Information on the tea samples.
