# Supplementary material for: Comparative Analysis of Volatile Compounds in Tieguanyin with Different Types Based on HS–SPME–GC–MS
Source: Foods. 2022 May 24;11(11):1530. doi: 10.3390/foods11111530 (PMC9180349; doi:10.3390/foods11111530)
Supplement: Supplementary file 1 [file foods-11-01530-s001.zip › foods-1721871-supplementary.pdf]

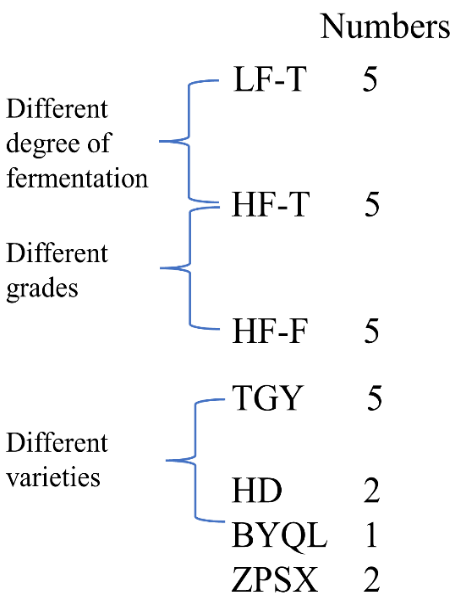

Figure S1. Information on the tea samples. LF-T: special-grade *Tieguanyin* with low fermentation, HF-T: special-grade *Tieguanyin* with heavy-fermentation, HF-F: first-grade *Tieguanyin* with heavy-fermentation, TGY: *Tieguanyin*, HD: *Huangdan*, BYQL: *Baiyaqilan*, and ZPSX: *Zhangpinshuixian*.
